# Monoclonal immunoglobulin measurement by mass spectrometry in patients with multiple myeloma and kidney failure: Analysis from the EuLITE trial

**DOI:** 10.1111/bjh.20168

**Published:** 2025-05-25

**Authors:** Graham McIlroy, Hannah V. Giles, Amy Rollins, Gemma Malin, Libby Jones, Nicola J. Wright, Oscar Berlanga, Simon North, Mark Cook, Colin Hutchison, Nils Heyne, Katja Weisel, Jennifer Pinney, Stephen Harding, Paul Cockwell, Guy Pratt

**Affiliations:** ^1^ University of Birmingham Birmingham UK; ^2^ University Hospitals Birmingham Birmingham UK; ^3^ The Binding Site (part of Thermo Fisher Scientific) Birmingham UK; ^4^ Bristol Myers Squibb Boudry Switzerland; ^5^ Hawke's Bay District Health Board Hastings New Zealand; ^6^ Universität Tübingen Tübingen Germany; ^7^ Universitätsklinikum Hamburg‐Eppendorf Hamburg Germany

**Keywords:** mass spectrometry, multiple myeloma, renal medicine

To the Editor,

Accurate and sensitive monitoring of monoclonal immunoglobulin is essential to assess treatment response in myeloma; however, there are significant challenges in patients with renal impairment. Here, we use mass spectrometry and demonstrate the greater accuracy of results in this important clinical setting.

Current methods for measuring serum free light chains (SFLC) do not differentiate between polyclonal and monoclonal free light chain (FLC), but rely on a skewed ratio to imply an underlying clonal protein. This leads to challenges in identifying low‐level pathological FLC, particularly in patients with severe renal impairment, due to complex alterations in the clearance of kappa and lambda light chains. There is uncertainty about the most appropriate reference ranges to apply in patients with severe renal impairment, and modified ranges have been proposed.^1^, ^2^ The most recent suggested modifications, based on results on the iStopMM study,^2^ require further validation.

Mass spectrometry (MS)‐based assays are more sensitive than serum protein electrophoresis and immunofixation (IFE), and nephelometric quantification of SFLC.^3^, ^4^ Characterising the specific mass‐to‐charge (*m*/*z*) value and isotype of pathological monoclonal proteins means they can be identified against a background of high polyclonal or oligoclonal immunoglobulin, and non‐pathological oligoclonal patterns can be more accurately identified post autologous stem cell transplant (ASCT). MS following immunopurification is recommended as an option for the diagnosis and monitoring of plasma cell disorders by the International Myeloma Working Group,^4^ but no studies have specifically assessed its utility in patients with plasma cell disorders who have significant renal impairment.

The aim of this study was to explore the accuracy of matrix‐assisted laser desorption/ionisation time‐of‐flight (MALDI‐TOF) MS for the measurement of monoclonal immunoglobulins in patients with multiple myeloma and renal impairment, by analysing available follow‐up samples from patients recruited to the prospective EuLITE trial. This work investigates the limitations of conventional disease assessment methods and explores the reasons for discordant results obtained by MS.

EuLITE (ISRCTN45967602, conducted in accordance with the Declaration of Helsinki, with national ethics approvals and patient consent) was a randomised controlled trial recruiting patients with newly diagnosed multiple myeloma and dialysis‐dependent acute kidney injury (AKI) due to myeloma cast nephropathy.^5^ All patients received the same induction chemotherapy (bortezomib, doxorubicin and dexamethasone) and were randomised between high‐cut‐off and high‐flux haemodialysis modalities. The primary outcome of dialysis independence at 90 days was not different between the two groups.^6^ Available serum samples from baseline and 3, 6 and 12 months post‐randomisation were included in this post hoc analysis. Patients were eligible for this study if they had sufficient stored serum from baseline and at least one follow‐up sample. Responses were categorised according to international consensus criteria,^7^ except complete responses which were unconfirmed (uCR) as bone marrow biopsy was not mandated. The available sample time point representing the best response was determined by the original trial data. Available serum samples were analysed using the EXENT® Immunoglobulin Isotypes (GAM) Assay for the EXENT Analyser (The Binding Site, part of Thermo Fisher Scientific, Birmingham UK).^8^ Follow‐up samples were deemed positive for persistent monoclonal protein when the EXENT software identified a peak of the same isotype and *m*/*z* (±4) as baseline. An MS‐based assay currently under development (FLC‐MS) specifically measures FLC and was also performed in all samples. FLC‐MS mass spectra were analysed manually by trained operators using FlexAnalysis (Bruker, Billerica, MA). Total immunoglobulin G (IgG), immunoglobulin A (IgA) and immunoglobulin M (IgM) concentrations were quantified with isotype specific reagents and SFLC concentrations with Freelite, using the Optilite system (The Binding Site). The reference range for SFLC ratio was adjusted primarily according to the values derived from the iStopMM study.^2^

The characteristics of the 37 patients included in this study are shown in Table 1. All baseline immunoglobulin isotypes identified using EXENT were consistent with the EuLITE trial data, including 2/37 (5%) patients who had biclonal disease. At the time of best response, 16/37 patients (43%) remained on dialysis and a further 10/37 patients (27%) had an estimated glomerular filtration rate (eGFR) ≤30 mL/min/1.73 m^2^.

**TABLE 1:** Patient characteristics.

Total number	37
Age, years	
Median (range)	63 (47–81)
Sex, *n* (%)	
Male	19 (51%)
Female	18 (49%)
Race, *n* (%)	
White	33 (89%)
South Asian	3 (8%)
Black	1 (3%)
eGFR at baseline, mL/min/1.73 m^2^	
Median (range)	6 (2–21)
Myeloma type, *n* (%)^a^	
IgG	11 (30%)
IgA	5 (14%)
IgD	2 (5%)
Light chain	21 (57%)
LC isotype, *n* (%)^a^	
Kappa	17 (46%)
Lambda	23 (63%)
Involved free light chain concentration, mg/L	
Median (range)	9489 (1200–61 900)
Dialysis allocation, *n* (%)	
HCO	15 (41%)
HF	22 (59%)
Received ASCT	
Number (%)	14 (38%)
Time to ASCT (months)	
Median (range)	9 (6–26)
Best response, *n* (%)	
Progressive disease	1 (3%)
Stable disease	2 (5%)
Partial response	11 (30%)
Very good partial response	16 (43%)
Complete response (unconfirmed)	7 (19%)
eGFR at best response	
Dialysis‐dependent, number (%)	16 (43%)
Median, mL/min.1.72 m^2^	33 (8‐106)

Abbreviations: ASCT, autologous stem cell transplant; eGFR, estimated glomerular filtration rate; HCO, high‐cutoff; HF, high‐flux; IgA, immunoglobulin A; IgG, immunoglobulin G; IgM, immunoglobulin M; LC, light chain.

^a^ Two patients had biclonal disease.

Best response data were obtained for all 37 patients: 14 patients (38%) from the 3‐month follow‐up time point, 8 patients (22%) at 6 months and 15 patients (41%) at 12 months; trial responses were compared with EXENT data (Figure 1). Of the 30 patients not in uCR, 26 had residual monoclonal protein detectable by EXENT, representing concordant response assessments. Four patients were EXENT‐negative but were labelled as IFE‐positive; three of these samples were following ASCT. For all four of these patients, EXENT identified oligoclonal immunoglobulins, none of which shared the same isotype and *m*/*z* as the baseline pathological monoclonal protein, indicating false positivity using conventional response data (Figure 1A). In five of the seven patients classified as uCR using conventional techniques, EXENT was positive (four with persistent monoclonal light chains, one with quantifiable monoclonal IgG‐kappa) (Figure 1B).

**FIGURE 1:**
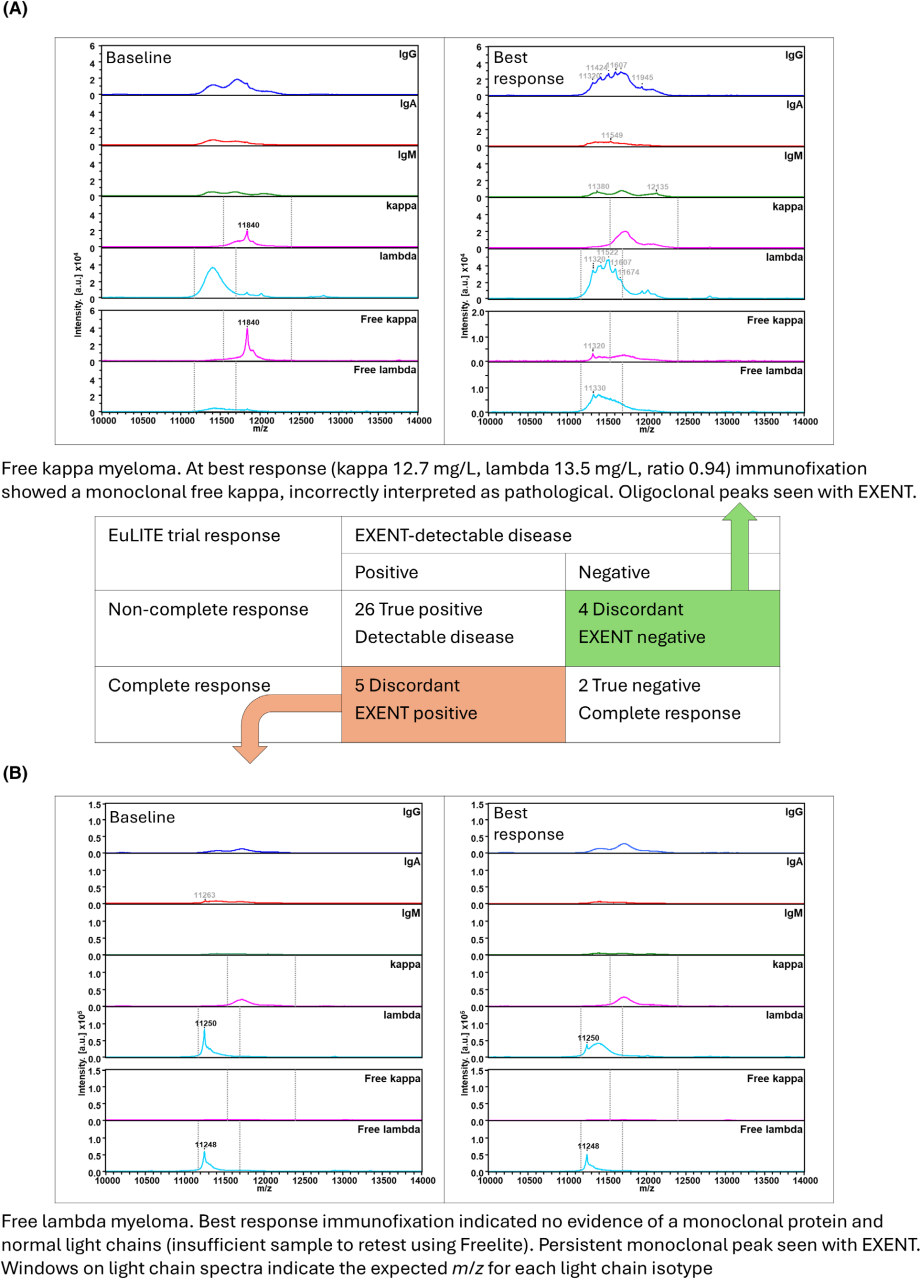
Illustrative spectra from discordant response assessments. (A) Immunofixation positive (very good partial response), EXENT shows no monoclonal protein. (B) Immunofixation negative (uCR), EXENT shows monoclonal protein. IgA, immunoglobulin A; IgG, immunoglobulin G; IgM, immunoglobulin M.

For 33 patients with sufficient sample volume at the time of best response, samples were re‐tested with Freelite to confirm the SFLC concentrations and results compared to FLC‐MS (Table S1). All 19 patients with a skewed SFLC ratio according to Freelite at best response also demonstrated a monoclonal light chain on EXENT and FLC‐MS. Fourteen had a normal SFLC ratio; however, for nine of these patients, FLC‐MS demonstrated persistent pathological immunoglobulin, including five categorised as achieving uCR and four with very good partial response (VGPR) due to persistent IFE positivity.

This study has used the more recent iStopMM‐defined renal reference ranges for SFLC ratio.^2^ An alternative renal reference range, derived from a smaller number of patients with dialysis‐dependent AKI, could have been used.^1^ This would have affected one patient with an eGFR of 34 mL/min/1.73 m^2^, changing an SFLC ratio of 0.38 from abnormal (using the iStopMM reference) to normal (using the older range). FLC‐MS demonstrated persistent pathological lambda light chain, indicating the superiority of the iStopMM range in this instance, although the overall agreement between the two ranges is notable. Further validation of the iStopMM reference ranges, including in more diverse populations, is required before these are adopted into routine care.

FLC‐MS is an advance on the current EXENT assay, having an increased analytical sensitivity for pathological FLC. This higher resolution is particularly advantageous in certain clinical contexts, for example, amyloidosis or oligosecretory disease,^9^, ^10^ as the background signal from intact immunoglobulin‐associated light chains is removed. All FLC‐MS results concorded with those obtained from EXENT except one: A patient with IgA‐lambda myeloma had no monoclonal immunoglobulin detectable by EXENT but was positive using FLC‐MS. This patient's best response was uCR, according to the standard methods used during the trial.

The small number of patients in this substudy limits the interpretation of overall survival outcomes. Patients failing to achieve at least VGPR had the poorest survival; all had persistent disease according to EXENT. The overall survival of patients according to the persistence of monoclonal immunoglobulin at best response, detected by EXENT and FLC‐MS, is shown in Figure S1.

The primary outcome of the EuLITE trial was independence from dialysis at 90 days. In the subset of patients included in the present study, this outcome was associated with best overall response, with patients achieving CR or VGPR more likely to be dialysis independent than those with poorer responses (*χ* ^2^ test, *p* = 0.015). Renal recovery and myeloma response, including according to EXENT, did not completely overlap, and two patients with undetectable disease using EXENT, nonetheless, remained on dialysis. Renal recovery from myeloma cast nephropathy is more strongly influenced by the rate of pathological SFLC clearance than the depth of response^11^; EXENT and FLC‐MS, therefore, did not provide additional information here, and there was no difference in MS results between trial arms. Further exploration of the clinical advantage of rapid and complete light chain clearance across a range of levels of renal impairment, including to MS negativity, is an important area of future work.

This study demonstrates the superior accuracy of MS when measuring monoclonal immunoglobulins in patients with significant renal impairment. Standard methods consistently miss residual pathological immunoglobulins at low concentrations, and also mis‐identify patients as having persistent disease when non‐pathological oligoclonal immunoglobulins are mis‐attributed as monoclonal protein. In the discordant cases identified in this study, the spectra provided by the MS platforms demonstrate why MS is more accurate. A formal analysis of sensitivity, specificity and positive and negative predictive values is not possible in this work, due in part to the relatively small and experimental patient population. However, this study demonstrates that its high analytical sensitivity, including in patients with renal impairment, may see MS techniques redefining the ‘Gold Standard’ for monoclonal immunoglobulin detection. Whether there is an additional benefit of using FLC‐MS over EXENT remains an important area for future research.

Using a skewed SFLC ratio to infer the presence of clonal FLC is inadequate, and more than half of the patients whose ratio normalised during treatment had persistent disease by electrophoresis. This is in keeping with previous data showing that, in patients with newly diagnosed myeloma without renal failure, FLC‐MS is more strongly predictive of progression‐free survival than SFLC ratio,^12^ and FLC‐MS negativity is associated with improved organ response and overall survival in AL amyloidosis.^9^, ^13^

Although this study has focussed on patients with myeloma and renal impairment due to cast nephropathy, the ability of MS techniques to detect low‐level monoclonal immunoglobulins and FLC in patients with renal impairment is likely to have utility across the spectrum of plasma cell disorders. It may be particularly advantageous in plasma cell disorders more typically associated with low‐level monoclonal protein production, such as monoclonal gammopathies of renal significance and AL amyloidosis, and may have potential in aiding the earlier identification of these conditions in patients with renal impairment, which need to be explored in further studies.

## AUTHOR CONTRIBUTIONS

GMcI, HVG, AR, GMa, LJ, NJW, OB, SN and GP performed the data acquisition, analysis and interpretation. MC, CH, NH, KW and PC designed the clinical trial and acquired the samples. JP, SH, PC and GP oversaw the project. GMcI, HVG and NJW drafted the manuscript. All authors read and revised the manuscript and approved the final version. All authors were involved in revising the manuscript and approving the final version. All authors agree to be accountable for all aspects of the work.

## FUNDING INFORMATION

No specific funding was received for this substudy. The EuLITE trial was funded by Gambro, Janssen and The Binding Site.

## CONFLICT OF INTEREST STATEMENT

HVG—Research grant: The Binding Site/Thermo Fisher. Speaker fees: Pfizer, Janssen. AR, GMa, LJ, NJW, OB, SN, SH—Employee of Thermo Fisher. MC—Employee and stockholder of BMS. NH—Research Grant: Chiesi (to the Institution). Honoraria: Alexion, Astellas, AstraZeneca, Baxter, Boehringer Ingelheim, Janssen, Novartis, Sanofi Aventis. Advisory Board: Alexion, AstraZeneca, Baxter, Chiesi. KW—Research Grant: Abbvie, Amgen, BMS/Celgene, GSK, Janssen, Sanofi (to the Institution). Honoraria: Abbvie, Amgen, Adaptive Biotech, Astra Zeneca, Beigene, BMS, Celgene, Janssen, GSK, Karyopharm, Menarini, Novartis, Oncopeptides, Pfizer, Roche, Sanofi, Stemline, Takeda. Advisory Board: Abbvie, Amgen, Adaptive Biotech, Beigene, BMS, Celgene, Janssen, GSK, Karyopharm, Menarini, Novartis, Oncopeptides, Pfizer, Regeneron, Roche, Sanofi, Takeda. GP—Research grant: The Binding Site/Thermo Fisher. Advisory board: The Binding Site/Thermo Fisher. GMcI, CH, JP, PC—None.

## Supporting information


Table S1.
Figure S1.

## Data Availability

Data are available from the corresponding author on request.
