# Supplementary material for: Monoclonal immunoglobulin measurement by mass spectrometry in patients with multiple myeloma and kidney failure: Analysis from the EuLITE trial
Source: Br J Haematol. 2025 May 25;207(1):294–8. doi: 10.1111/bjh.20168 (PMC12234270; doi:10.1111/bjh.20168)
Supplement: Supplementary file 1 — Table S1. Figure S1. [file BJH-207-294-s001.docx]

**Supplementary table 1 - Freelite and FLC-MS comparison**

| **Best response** | **Best response timepoint (months)** | **Serun free light chain measurements** | | | **eGFR (mL/min/1.73m^2^)** | **FLC ratio abnormal** | | **Monoclonal protein detected** | |
| --- | --- | --- | --- | --- | --- | --- | --- | --- | --- |
|  |  | **Kappa (mg/L)** | **Lambda (mg/L)** | **Ratio** |  | **Hutchison ref range** | **iStopMM ref range** | **EXENT** | **FLC-MS** |
| **Concordant normal/abnormal FLC ratio and MS-negative/positive monoclonal protein** | | | | | | | | | |
| PR | 3 | 16.68 | 9543.22 | 0.002 | Dialysis | Abnormal | Abnormal | Positive | Positive |
| PR | 12 | 10.44 | 6719.88 | 0.002 | 29 | Abnormal | Abnormal | Positive | Positive |
| PR | 6 | 13.62 | 1708.95 | 0.008 | Dialysis | Abnormal | Abnormal | Positive | Positive |
| PR | 12 | 11.6 | 1385.56 | 0.008 | 8 | Abnormal | Abnormal | Positive | Positive |
| PR | 3 | 31.62 | 3404.23 | 0.009 | Dialysis | Abnormal | Abnormal | Positive | Positive |
| PR | 3 | 59.72 | 1253.19 | 0.048 | Dialysis | Abnormal | Abnormal | Positive | Positive |
| PD | 12 | 19.16 | 195.97 | 0.098 | 20 | Abnormal | Abnormal | Positive | Positive |
| VGPR | 12 | 13.01 | 86.22 | 0.151 | 36 | Abnormal | Abnormal | Positive | Positive |
| VGPR | 3 | 6.46 | 36.85 | 0.175 | 90 | Abnormal | Abnormal | Positive | Positive |
| VGPR | 12 | 56.88 | 231.4 | 0.246 | Dialysis | Abnormal | Abnormal | Positive | Positive |
| VGPR | 12 | 24.48 | 63.81 | 0.384 | 34 | Normal | Abnormal* | Positive | Positive |
| VGPR | 12 | 10.69 | 11.54 | 0.926 | 52 | Normal | Normal | Negative | Negative |
| VGPR*** | 12 | 12.72 | 13.52 | 0.941 | 106 | Normal | Normal | Negative | Negative |
| uCR | 3 | 17.91 | 12.48 | 1.435 | 19 | Normal | Normal | Negative | Negative |
| VGPR | 12 | 27.3 | 18.64 | 1.465 | Dialysis | Normal | Normal | Negative | Negative |
| VGPR | 12 | 17.3 | 10.65 | 1.624 | 26 | Normal | Normal | Negative | Negative |
| VGPR | 12 | 52 | 10.66 | 4.878 | 43 | Abnormal | Abnormal | Positive | Positive |
| PR | 3 | 933.65 | 56.48 | 16.531 | Dialysis | Abnormal | Abnormal | Positive | Positive |
| VGPR | 6 | 209.51 | 2.95 | 71.02 | 57 | Abnormal | Abnormal | Positive | Positive |
| PR | 3 | 674.88 | 7.25 | 93.087 | Dialysis | Abnormal | Abnormal | Positive | Positive |
| SD | 3 | 6776.38 | 14.06 | 481.962 | Dialysis | Abnormal | Abnormal | Positive | Positive |
| PR | 6 | 7726.55 | 11.23 | 688.028 | Dialysis | Abnormal | Abnormal | Positive | Positive |
| SD | 3 | 1637.15 | 1.89 | 866.217 | Dialysis | Abnormal | Abnormal | Positive | Positive |
| PR | 3 | 23036.6 | 2.67 | 8627.94 | 27 | Abnormal | Abnormal | Positive | Positive |
| **Discordant normal FLC ratio with MS-positive monoclonal protein** | | | | | | | | | |
| uCR | 3 | 14.43 | 24.41 | 0.591 | 25 | Normal | Normal | Positive | Positive |
| uCR | 3 | 8.2 | 13.4 | 0.612 | Dialysis | Normal | Normal | Negative | Positive** |
| VGPR | 6 | 22.7 | 34.21 | 0.664 | 10 | Normal | Normal | Positive | Positive |
| uCR | 6 | 17.34 | 20.28 | 0.855 | Dialysis | Normal | Normal | Positive | Positive |
| VGPR | 3 | 9.91 | 10.56 | 0.938 | 30 | Normal | Normal | Positive | Positive |
| uCR | 12 | 19.52 | 19.07 | 1.024 | 37 | Normal | Normal | Positive | Positive |
| VGPR | 12 | 26.6 | 21.21 | 1.254 | Dialysis | Normal | Normal | Positive | Positive |
| uCR | 6 | 151 | 85.92 | 1.757 | 33 | Normal | Normal | Positive | Positive |
| VGPR | 12 | 29.57 | 13.9 | 2.127 | 46 | Normal | Normal | Positive | Positive |

All patients with Freelite results, arranged in order of the kappa:lambda ratio, group according to consensus between abnormal free light chain ratio and mass spectroscopy-positive disease. FLC, free light chain; PR, partial response; PD, progressive disease; VGPR, very good partial response; uCR, unconfirmed complete response; SD, stable disease. *One patient with FLC ratio abnormal only using iStopMM reference range. **One patient with monoclonal immunoglobulin only detectable using FLC-MS. ***Patient included in 1A of Figure 1.

## Supplementary figure 1 – Overall survival

Overall survival of included patients, according to persistence of monoclonal immunoglobulin at best response timepoint, determined by (A) EXENT and (B) FLC-MS.


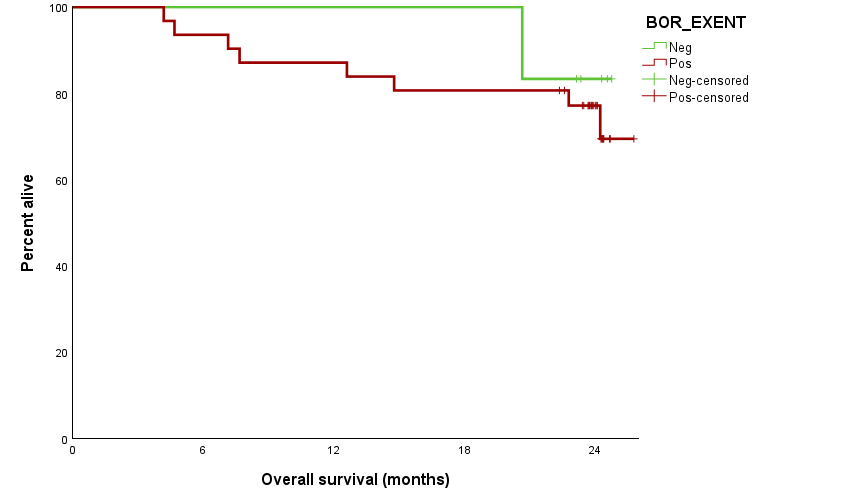


Number at risk:

6 6 6 6 3

31 29 27 25 13

EXENT negative for monoclonal protein

EXENT positive for monoclonal protein


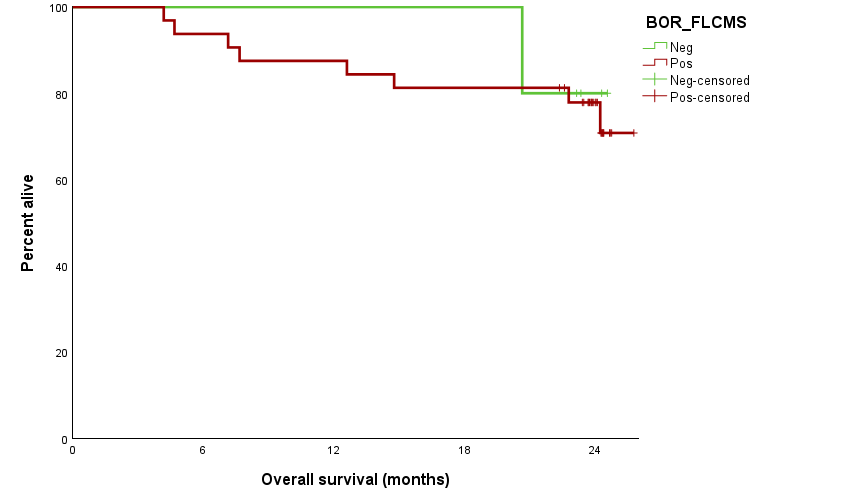


Number at risk:

5 5 5 5 2

32 30 28 26 14

FLC-MS negative for monoclonal protein

FLC-MS positive for monoclonal protein
